# Poly(3-hydroxybutyrate-CO-3-hydroxyvalerate) PHBHV biocompatible nanocarriers for 5-FU delivery targeting colorectal cancer

**DOI:** 10.1080/10717544.2019.1582729

**Published:** 2019-03-21

**Authors:** Ionut Cristian Radu, Ariana Hudita, Catalin Zaharia, Bianca Galateanu, Horia Iovu, Eugenia (Vasile) Tanasa, Sabina Georgiana Nitu, Octav Ginghina, Carolina Negrei, Aristidis Tsatsakis, Kelly Velonia, Mikhail Shtilman, Marieta Costache

**Affiliations:** a Advanced Polymer Materials Group, University Politehnica of Bucharest, Bucharest, Romania;; b Department of Biochemistry and Molecular Biology, University of Bucharest, Bucharest, Romania;; c University Politehnica of Bucharest, Bucharest, Romania;; d National Research and Development Institute for Chemistry and Petrochemistry – ICECHIM, Bucharest, Romania;; e Department of Surgery, Sf. Ioan Emergency Clinical Hospital, Bucharest, Romania;; f Department II, Faculty of Dental Medicine, Carol Davila University of Medicine and Pharmacy Bucharest, Bucharest, Romania;; g Department of Toxicology, Faculty of Pharmacy, Carol Davila University of Medicine and Pharmacy, Bucharest;; h Department of Toxicology and Forensic Sciences, Faculty of Medicine, University of Crete, Heraklion, Greece;; i Department of Materials Science and Technology, University of Crete, Heraklion, Greece;; j Department of Polymers, D.I. Mendeleyev University of Chemical Technology, Moscow, Russia

**Keywords:** Nanocarrier, 5-FU, polyhydroxyalkanoate, HT-29, colorectal cancer, anticancer efficacy

## Abstract

Aiming to address the issue of poor bioavailability of most anti-tumor medicines against colorectal cancer, we developed a targeted anticancer nanocarrier using biocarriers able to both bind and easily release their load in a controlled manner. Poly(3-hydroxybutyrate-co-3-hydroxyvalerate) carriers were obtained via the emulsification-diffusion method, loaded with 5-fluorouracil and then characterized in terms of particle morphology and size (SEM, DLS), drug uptake and release. The cytotoxic potential of the 5-fluorouracil-loaded polymer nanocarriers on human adenocarcinoma cells (HT-29 cell line) was investigated. The *in vitro* studies clearly demonstrated that while the nanocarriers themselves slightly alter HT-29 cell viability, when loaded with 5-fluorouracil they significantly decrease cell viability, suggesting that the polymer itself exhibits low cytotoxicity and the drug-loaded carrier acts in an efficient manner to kill HT-29 human adenocarcinoma cells.

## Introduction

Worldwide, an increasing risk for developing colorectal cancer is determined by aggravating factors such as, primarily, the occurrence of inflammatory bowel disease as well as the incidence of obesity and type 2 diabetes, all associated with additional consumption of heavily calorie-laden and highly processed food accompanied by the widely spread lack of exercise (Meyerhardt et al., 2003; Jemal et al., 2008; Khalili & Chan, 2012; Khalili et al., 2015).

Once diagnosed, colorectal cancer intervention strategies depend on the stage of the disease. Thus, localized forms of colon cancer, i.e. stage I–III may primarily be treated and potentially cured by surgical resection. In addition to surgery, therapy includes adjuvant chemo-therapy, considered standard for stage III colorectal cancer and still under debate for stage II disease (Krook et al., 1991; Zaheer et al., 1998; Lindsetmo et al., 2008; Matsuda et al., 2018).

Among several such types of cytotoxic chemotherapy, the first to be implemented for over four decades, has been 5-fluorouracil (5-FU) and leucovorin, leading to notable improvement in overall survival rate as compared to control groups. In this context, and with surgery proving less effective for metastatic colorectal cancer, chemotherapy has become more a standard therapeutic approach (Arkenau et al., 2003; André et al., 2004; Pardini et al., 2011; He et al., 2017; Wang et al., 2017).

Characterized by nanosized dimensions and engineered specificity, nanomedicines can be tailored to include multiple component drugs *per se* combined in customized drug delivery systems addressing the complicated needs of chemotherapy. Development of colloidal nanoparticles consisting of polymers, dendrimers, lipids, as well as organometallic and carbon-based materials, with sizes less than a micron for anticancer drug targeting contemplates using carriers able to both bind and easily release their load in a controlled manner (Torchilin, 2010; Bartoş et al., 2016; Quinn et al., 2017; Hunter & Moghimi, 2017; Kuskov et al., 2017; Viard et al., 2018; Zhao & Stenzel, 2018; Taghizadehghalehjoughi et al., 2018; Luss et al., 2018). Polymer nanocarriers, in particular, have been at the focal point of targeted and controlled drug delivery studies since their tunable chemistry allows to adjust biocompatibility, toxicity, size, surface chemistry, and stability in biological systems with the aim to improve the efficacy of drugs with physicochemical (e.g. poor solubility) or toxicity barriers (Bartoş et al., 2016; Jain et al., 2016; Katiyar et al., 2016). Extensive research during the recent decades has brought several such nanomedicines into everyday clinical practice while over twelve nanoparticles for diverse therapies, cancer included, are currently under various stages of development (Neagu et al., 2016; Piperigkou et al., 2016; Neagu et al., 2017; Engin et al., 2017).

The intracellular uptake capacity of polymeric nanovehicles is highly influenced by their size, shape, and surface chemistry which are versatile and often stabilized by surfactants or tuned by hydrophilic surface modification leading to stealth-based formulations.

Micro- and nanoparticles are solid colloidal systems often stabilized by surfactants at the particle/water interface. Their most important characteristics with high influence over intra-cell uptake capacities are size-range, shape, and surface chemistry while hydrophilic surface modification of such particles often leads to stealth formulation (Zhang et al., 2016). Capsules, in particular, are vesicular systems with a typical core-shell structure where the core can be a polymeric reservoir and/or an inner liquid and the shell surrounding the core can be a polymeric membrane or coating. The drug is usually loaded within the core by either dissolution in the inner liquid or by dispersion in the polymeric reservoir but may also be adsorbed on the capsule surface. Spheres, on the other hand, have a matrix type structure comprising a unique polymer, without inner core or surrounding shell. In this case, the drug can be dispersed within a polymeric matrix or adsorbed to the sphere surface (Cheng et al., 2007; Mora-Huertas et al., 2010).

Considering that the active substance can be either entrapped inside or adsorbed on the surface of both types of particles, drug loading is mostly achieved via two methods: either by incorporating the drug during particle preparation or by adsorbing the drug on the surface of preformed particles. In the first case, the drugs are added in the polymer solution or even in the reaction mixture during the polymerization process. In the second case, the active substances can be loaded by incubation with the microparticle solution (Pinto Reis et al., 2006). Apart from particle size, drug carriers are also characterized by size distribution, surface charge, surface adhesion, and interior porosity all affecting drug encapsulation efficiency and stability. It has been shown that surface charge and chemistry are also very important for the interaction with blood components such as proteins, antibodies, or small molecules, as well as for the adherence and interaction with cell membranes (Gratton et al., 2008; Ferreira & Trierweiler, 2009; Kumari et al., 2010). Biodegradable polymers have been in the focus of state-of-art studies for obtaining pharmaceutic polymeric nanoparticles encapsulating a variety of therapeutic substances due to their capacity of being biodegraded by the body (Casarin et al., 2011; Leong et al., 2018). In this direction, poly(hydroxybutyrate-co-hydroxyvalerate) (PHBHV), a natural polyhydroxyalkanoate produced from renewable sources by diverse microorganisms, presents significant interest. PHBHV is a copolymer of the well-studied poly(3-hydroxybutyrate) (PHB) used in numerous medical studies with applications such as implants for tissue engineering due its biocompatibility and biodegradability. Importantly, literature reports demonstrate no secondary reactions causing acute inflammation or tissue necrosis in tissue adjusted to PHBHV implants. Furthermore, PHBHV shows great potential for obtaining biomedically important micro- and nanocarriers as it is highly soluble in organic solvents such as chloroform or dichloromethane and has poor solubility in all other solvents. Due to this poor solubility in most solvents, micro- and nanoparticles can be easily obtained via the emulsification-solvent evaporation method, or even via the nanoprecipitation method (Pich et al., 2006; Poletto et al., 2007). Recently, spherical PHBHV nanoparticles were found capable of delivering Silymarin into HT-29 colon cancer cells without cytotoxic effects or influence on cell viability (Radu et al., 2017).

The research presented herein aimed at modeling a new drug delivery carrier with well-defined size and morphology for the delivery of 5-FU in colorectal adenocarcinoma cells. We focused on the obtaining and characterizing PHBHV particles decorated with a hydrophilic polyvinyl alcohol surface to yield core-shell structures via optimizing a well-established emulsification-diffusion method. We report on the effect of the solute concentrations on the diameter, size distribution and shape morphology by dynamic light scattering (DLS) and scanning electron microscopy (SEM) of PHBHV-PVA nanocarriers. Based on these studies, the appropriate protocol to develop the desired nanocarriers was elucidated, while 5-FU was directly loaded during particle formation by dissolution into the stabilizer aqueous phase. Importantly, during drug release studies, the *in vitro* anticancer efficacy of 5-FU loaded nanoparticles was evaluated against HT – 29 human colon cancer cells after 2, 6, and 24 h.

## Materials and methods

Poly(3-hydroxybutyrate-co-3-hyroxyvalerate) (PHBHV) with a molecular weight of 67,000 g/mole containing 2% hydroxyvalerate was obtained from Good Fellow. Polyvinyl alcohol (PVA) with a molecular weight of 88,000 g/mole, 88% hydrolyzed, 5-fluorouracil (5-FU) and chloroform were provided by Sigma-Aldrich.

### Poly(3-hydroxybutyrate-co-3-hydroxyvalerate) particles preparation and 5 – fluorouracil loading procedure

**Figure 1.**
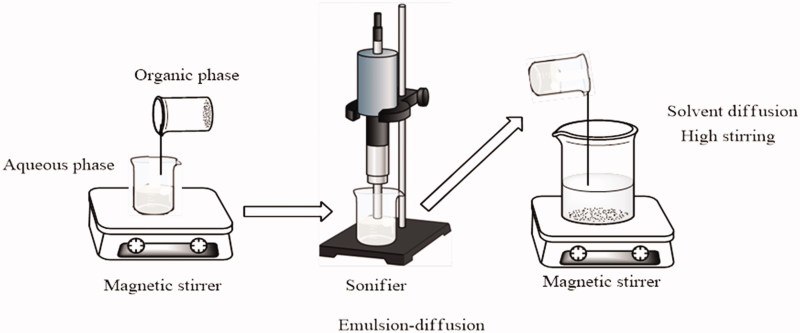
Set-up used for the preparation of particles via the emulsion-diffusion method.

The protocol was optimized from literature methods (Casarin et al., 2011; Leong et al., 2018) to obtain particles with full control over their size by varying the concentrations of the solutes in the organic and aqueous phase, containing PHBHV and PVA, respectively. Briefly, a fixed concentration of PHBHV solution in chloroform was prepared by stirring under reflux at 62 °C for 6 h to enable better dissolution of the polymer in the solvent (Figure 1). The homogenous polymer solution was slowly added under vigorous stirring to a fixed concentration solution of PVA in distilled water, maintaining a 1:10 v/v final ratio. The resulting suspension was subsequently sonicated to achieve high dispersion of the polymer in the aqueous phase and then transferred to distilled water to form a 1:5 (v/v) solution from which, the organic solvent was evaporated under reduced pressure. After centrifugation, the recovered particles were extensively washed with ultrapure water to remove residual PVA traces.

To study the influence of polymer (organic phase) and stabilizer concentration (aqueous phase) on particle size and size distribution, various concentrations of the two phases were screened. Different formulations were prepared by varying either the polymer concentration in the range of 1–5% w/w while the other parameters were kept constant, or by similarly varying the stabilizer concentration in the same range.

Considering the excellent solubility of 5-FU into the PVA solution, the drug encapsulation was performed by directly adding 5-FU in the stabilizer PVA solution at a final concentration of 0.2 mg/ml, followed by one-day dissolution. Drug-loaded particles were obtained by dropwise addition of PHBHV solution into the stabilizer solution containing the previously dissolved drug. The obtained drug-loaded nanoparticles had a final drug content of ∼2 mg of drug/g of the nanoparticle. The drug encapsulation was performed only for nanoparticles obtained from 2% polymer concentration.

### Evaluation of 5-FU encapsulation efficiency and in vitro release

The amount of 5-FU incorporated in the PHBHV particles was investigated by UV-Vis spectrophotometry based on the 5-FU unique absorbance peak in the UV-Vis region at 265 nm. To determine the 5-FU content, the UV-VIS absorbance was measured at 265 nm using a Shimadzu UV-Vis-NIR spectrophotometer (Kyoto, Japan). The encapsulation efficiency (EE) was determined using the following equation:

$$\mathrm{EE}(\%)=\frac{\mathrm{amount}\;\mathrm{of}\;\mathrm{the}\;\mathrm{loaded}\;5\;-\;\mathrm{FU}}{\mathrm{total}\;\mathrm{amount}\;\mathrm{of}\;\mathrm{the}\;5-\mathrm{FU}}\times 100$$

The release of 5-FU from PHBHV nanoparticles was studied in phosphate buffer saline solution (PBS) for 48 h in a controlled environment. The obtained drug-loaded particles solution was entrapped in a cellulose membrane, immersed in 40 mL of PBS (0.01 M, pH 7.45) and incubated in a precision water bath (orbital mixer Benchmark Scientific, Sayreville, NJ) at 400 rpm and 37 ^°^C. Aliquots (5 mL) containing a mixture of PBS and released 5-FU were collected at defined time points and the release medium was refreshed with the addition of an equal amount of fresh PBS after each withdrawal in order to maintain the total volume of the sample constant. Drug release was studied for 48 h and the aliquots were collected every 15 min in the first hour, every 30 min in the next 3 hours and every 60 min until the end of the predetermined time interval. The 5-FU release profile from nanoparticles was evaluated by UV-Vis spectroscopy.

### Dynamic light scattering, zeta potential and scanning electron microscopy (SEM)

The size distribution and zeta potential were investigated by Dynamic Light Scattering (DLS) using a Zetasizer Malvern DLS device (Malvern, United Kingdom) for particles obtained from 2% w/v polymer concentration.

The size and size distribution of the carriers, as well as their morphology, was evaluated by SEM using a Quanta Inspect F Scanning Electron Microscopy (Waltham, MA) device equipped with 1.2 resolution field emission gun (FEG) and an X-ray energy dispersive spectrometer (EDS).

### In vitro cytotoxicity assessment of PHBHV nanocarriers

#### Cell culture model

The HT – 29 human colon adenocarcinoma cell line (ATCC – American Type Culture Collection) was used as a cellular model in this study. Cells were cultured as monolayers at 37 °C under a humidified atmosphere of 5% CO_2_, in Dulbecco’s modified Eagle`s Medium (DMEM) supplemented with 10% fetal bovine serum (FBS) and a 1% antibiotic – antimitotic formulation containing a mixture of penicillin and streptomycin. The medium renewal was carried out 2–3 times/week. When the confluence reached 80%, cells were routinely subcultured using enzymatic treatment with trypsin/EDTA solution for cellular detachment from culture vessels.

To *in vitro* evaluate the cytotoxicity of the drug-loaded nanocarriers, HT – 29 colon cancer cells were seeded at an initial density of 2 × 10^3^ cell/cm^2^ on flat bottom 96-well plates. These cultures were left to adhere 24 h prior treatment and the cell attachment was confirmed in brightfield microscopy using an inverted Nikon microscope (Amsterdam, Netherlands).

#### Cellular viability assessment by MTT assay

The MTT assay was employed to evaluate the HT – 29 colon cancer cells viability after exposure to drug-free PHBHV nanoparticles or 5-FU loaded PHBHV nanoparticles. Briefly, the HT – 29 colon cancer cells monolayers were treated for 2, 6, and 24 h with 5-FU (5 mM), drug-free and 5-FU loaded PHBHV carriers. At each fixed experimental time point, the culture medium was discarded, and the monolayers were thoroughly washed with PBS. The samples were next incubated for 4 h at 37 °C in a 1 mg/ml MTT freshly prepared solution in serum-free culture media. The formed formazan crystals were consequently dissolved in DMSO and the absorbance of the resulting solutions was measured at 550 nm using a FlexStation III (Molecular Devices, San Jose, CA) spectrophotometer. An untreated control was prepared following the same procedure and used as a reference.

#### PHBHV nanocarriers cytotoxicity potential on HT – 29 Colon cancer cells

The cytotoxic potential of the 5-FU loaded PHBHV nanoparticles on HT – 29 colon cancer cells was investigated by spectrophotometric evaluation of Lactate Dehydrogenase (LDH) activity in the culture media. Briefly, after 2, 6, and 24 h of treatment with 5-FU (5 mM), drug free and 5-FU loaded PHBHV nanocarriers, the culture medium was collected, mixed with the components of the *In Vitro* Toxicology Assay Kit (TOX-7 kit, Sigma Aldrich, Saint Louis, MO) according to the manufacturer`s instructions and incubated in darkness at room temperature for 20 min. After time expired, the absorbance of the samples was recorded at 490 nm using a FlexStation III (Molecular Devices) spectrophotometer. As a control, culture medium harvested from untreated monolayers was used and processed under the same protocol.

## Results

### Preparation and morphological characterization of PHBHV particles (SEM)

**Figure 2.**
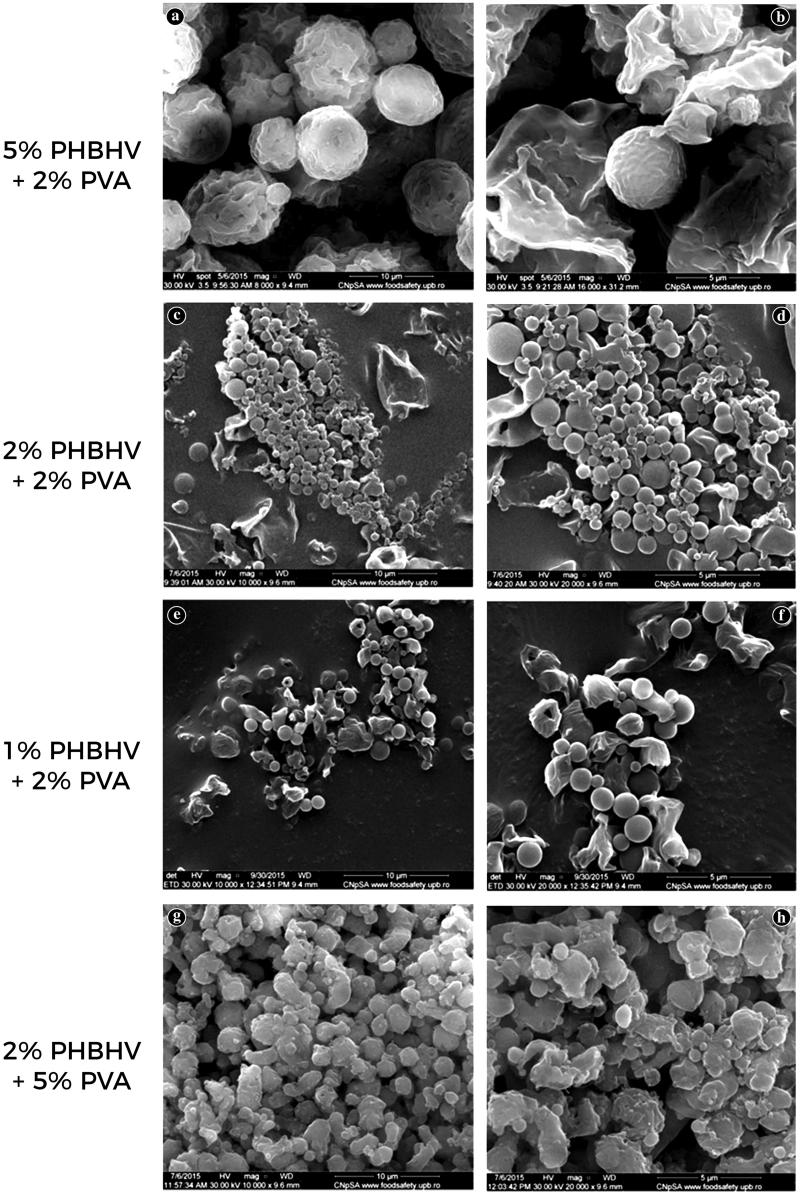
SEM micrographs of the PHBHV particles prepared under different experimental conditions.

New core-shell PVA-PHBHV particles were obtained by two emulsification-diffusion steps with varying polymer phase concentration to ensure control over particle size. A comparative screening of the effect of polymer and stabilizer concentration to the morphology of the resulting nanoparticles was performed to establish the optimal polymer to stabilizer ratio for obtaining the appropriate-sized PHBHV particles (not all data are shown). SEM investigation proved that the ratio of concentrations in the organic and aqueous phase during particle preparation had a strong impact on the size and shape of the obtained particles. As it can be shown in Figure 2(a), when using a 5% w/v PHBHV solution (50 mg/ml) and a 2% w/v PVA solution (20 mg/ml), microparticles in the range of microns (1–7 microns) with large polydispersity were obtained. Additionally, their shape was found to be irregular probably due to residual PVA that functions both as coating and as a stabilizer agent. At higher magnification (Figure 2(b)) it can be clearly observed that the particles are trapped in a mass that can probably be attributed to the stabilizer and therefore several additional washing steps need to be performed to remove the residual PVA traces (Galindo-Rodriguez et al., 2004). Furthermore, a 5% w/v concentration of PHBHV led to the formation of a small relative number of particles possessing the largest size observed under all the tested conditions, indicating that a high polymer concentration can easily influence both size and number of the synthesized particles.

A larger relative number of nanoparticles with a significant decrease in size was obtained by decreasing the polymer concentration. Using a 2% w/v PHBHV solution (20 mg/ml) and a 2% w/v stabilizer (PVA) concentration, the formation of particles with sizes varying between 100 nm and 1 µm was observed. The SEM micrographs (Figure 2(c,d)) revealed a wide distribution for the particles possessing spherical shape and a clean surface. More importantly, using the above mentioned experimental conditions, a large relative number of well-individualized particles was obtained, clearly not entrapped in the stabilizer mass as observed at the preparations using high concentrations of polymer.

To obtain smaller particles, the concentration of PHBHV was decreased to 1% w/v (10 mg/ml), maintaining a 2% w/v concentration for PVA. As shown in Figure 2(e,f), the particles obtained for this set of parameters possessed narrower size distribution of around 700 nm–1 µm. Furthermore, the micrographs revealed that the obtained nanoparticles were well individualized and possessed a round shape morphology characterized by a clean surface. The relative number of particles and their characteristics were compared with those obtained using a 2% concentration of PHBHV, and did not present a decrease in size probably since the 2% w/v polymer concentration represents an equilibrium concentration and under this limit, smaller particles cannot be obtained.

To highlight the influence of the stabilizer concentration upon the microparticles size and shape, the PVA concentration was varied between 1% and 5%. The use of a high concentration of 5% w/v PVA (50 mg/ml) combined with a low concentration of polymer (2% w/v) led to an increase in the size of the synthesized particles, varying between 1 and 3 microns (microparticles). As observed in the SEM micrographs (Figure 2(g,h)), under these experimental conditions the particles lose their spherical shape and are clearly trapped in the stabilizer mass, while the final quantity of the obtained particles is not affected. Thus, the stabilizer concentration influences only the size and shape of the particles presumably due to the accumulation of excess PVA on the particle surface.

### Dynamic light scattering and zeta potential

**Figure 3.**
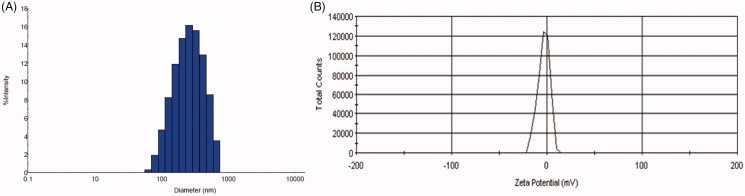
(A) Size distribution by DLS for the 2% unloaded PHBHV nanoparticles; (B) Zeta potential for the 2% unloaded PHBHV particles.

To obtain better characterization and more accurate measurement of the 2% PHBHV particles, the size distribution in water was investigated by DLS. The size distribution profile of the unloaded 2% PHBHV particles revealed one peak (Figure 3(A)) attributed to the PHBHV nanoparticles and a mean size of around 420 ± 10 nm with a polydispersity index (PDI) of 0.39% fitting a polydisperse unimodal model.

Moreover, the zeta potential of the PHBHV nanoparticles was calculated since the surface charges of the particles can offer valuable information regarding the interaction with cells, plasma proteins or dimensional stability. The zeta potential of the 2% PHBHV nanoparticles was estimated at −2.31 mV, which is good as also supported by the SEM micrographs showing well-individualized round nanoparticles with no aggregates. Furthermore, nanoparticles with a close-to-neutral zeta potential, in general, show good cellular uptake and less nanotoxicity for cells than nanoparticles with higher zeta potential values (Figure 3(B)).

### Release of 5-FU from PHBHV nanoparticles

**Figure 4.**
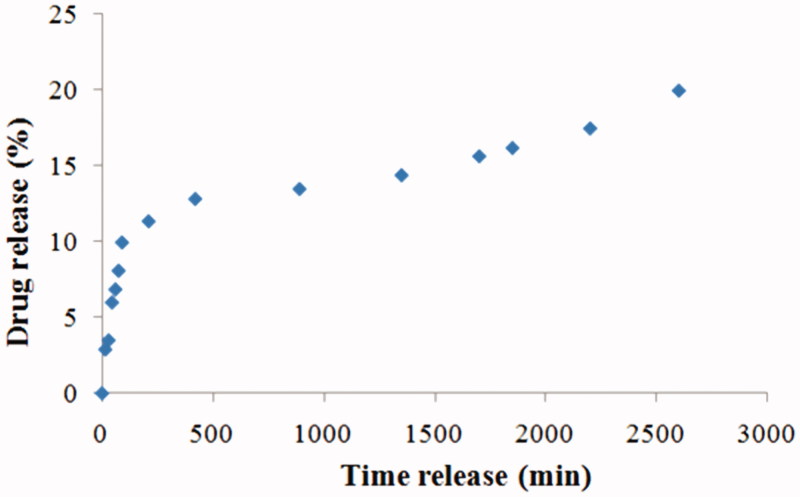
*In vitro* release profile of 5-fluorouracil from drug loaded 2% PHBHV nanocarriers.

The drug release rate study for the 5-FU loaded 2% PHBHV nanoparticles in PBS (Figure 4) revealed that 20.1% of the total encapsulated 5-FU was released during the first 42 h. The drug release rate profile further revealed fast drug release during the first 6 h and a slower release for the next 36 h. The different time-dependent release profiles can be attributed to the release of 5-FU molecules entrapped on different parts of the microparticles comprising a stabilizer shell and a polyester core. More specifically, drug adsorbed on the surface of the particles surface can be expected to exhibit fast release, while drug entrapped within the stabilizer shell near the surface will manifest a slower release. The PHBHV core of the particles can also entrap the drug with an even slower release depending on biodegradation rate of the polyester (Gombotz & Pettit, 1995; Saito et al., 2005; Kwon & Furgeson, 2007). Upon isolation of the nanocarriers according to the protocol, the water phase was recovered and evaluated by UV-VIS to reveal the concentration of drug that was not incorporated in the nanocarriers. The results showed that 65% of initial drug amount remained into the water phase indicating that ∼35% of the initial drug amount was loaded into the nanocarriers resulting in an encapsulation efficiency of about 35%.

### Effect of treatment with unloaded and 5-FU loaded PHBHV nanocarriers on HT – 29 cells viability

**Figure 5.**
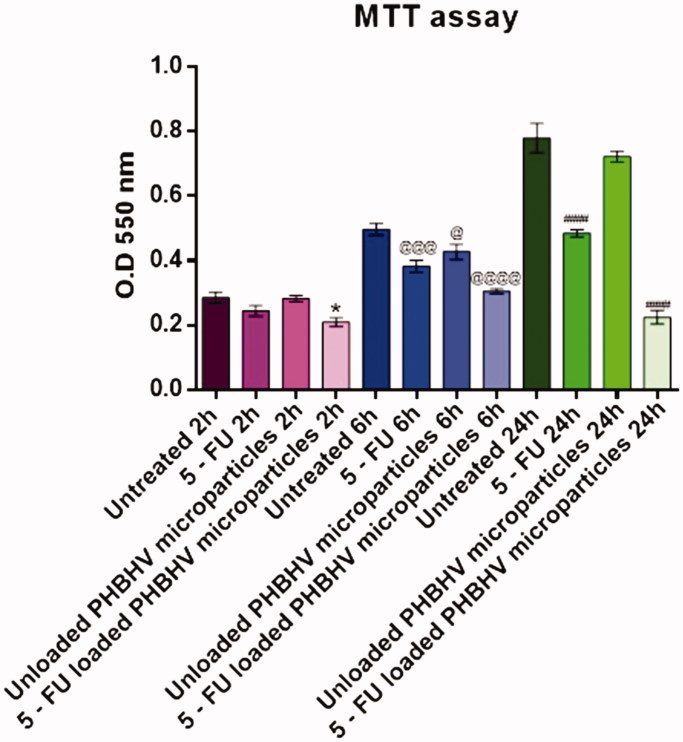
Graphical representation of MTT viability data (*p < .05 5-FU loaded PHBHV nanoparticles vs. untreated sample after 2 h; @*p* < .05 unloaded PHBHV particles vs. untreated sample after 6 h; @@@*p* < .001 5-FU vs. untreated sample after 6 h; @@@@*p* < .0001 5-FU loaded PHBHV nanoparticles vs. untreated sample after 6 h; ###*p* < 0.001 5-FU vs. untreated sample after 24 h; ####*p* < .0001 5-FU loaded PHBHV nanoparticles vs. untreated sample after 24 h.

To evaluate the viability of HT – 29 human colon cancer cells after 2, 6, and 24 h of exposure to free 5-FU, unloaded PHBHV nanoparticles, and 5-FU loaded PHBHV nanoparticles, the quantitative MTT assay was performed. Data were statistically analyzed and graphically represented in Figure 5. After 2 h of treatment, no significant differences were observed between the 5-FU and the unloaded PHBHV nanocarriers treated cells as compared to an untreated control. However, treatment with 5-FU loaded PHBVH nanoparticles slightly decreased the cellular viability (*p* < .05) as compared with that of the untreated cells. After 6 h of exposure, all the treatment regimens affected cellular viability, the strongest effect being observed for 5-FU treatment (*p* < .001) and PHBHV 5-FU loaded nanoparticles (*p* < .0001). Interestingly, exposure to bare PHBHV particles also induced changes in HT – 29 cell viability (*p* < .05), showing that the polymer itself expresses in time its own toxicity upon HT – 29 colon cancer cells. Moreover, after 24 h under treatment with 5-FU and 5-FU loaded PHBHV carriers the cellular viability dramatically decreased (*p* < .0001) as compared with the reference. In contrast, no significant changes in the cellular viability of HT – 29 cells exposed to unloaded PHBHV nanoparticles were observed at this time point, showing that the polymer induces a moderate toxicity at the beginning, that is not further increased.

### Evaluation of PHBHV nanocarriers cytotoxic potential

**Figure 6.**
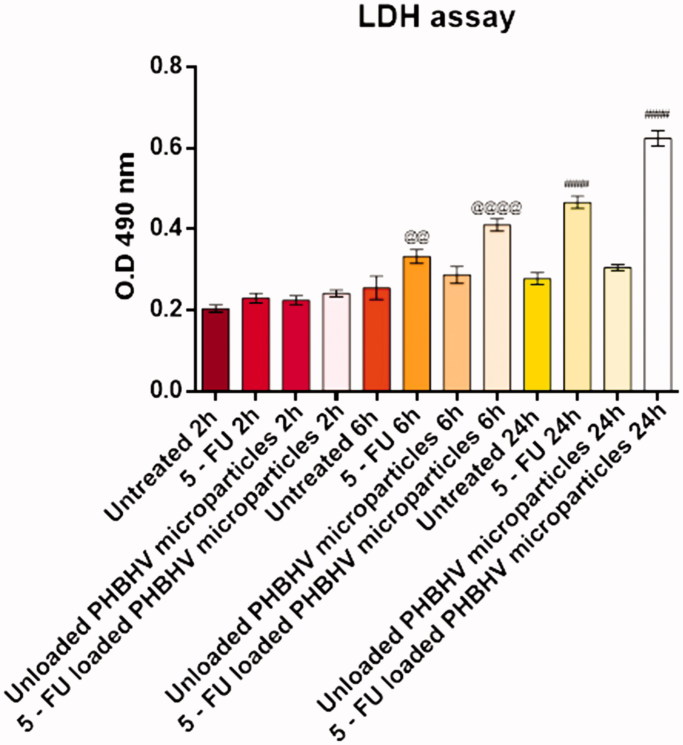
Graphical representation of LDH cytotoxicity data (@@*p* < .001 5-FU vs. untreated sample after 6 h; @@@@*p* < .0001 5-FU loaded PHBHV nanoparticles vs. untreated sample after 6 h; ####*p* < .0001 5- FU vs. untreated sample after 24 h; ####*p* < .0001 5-FU loaded PHBHV nanoparticles vs. untreated sample after 24 h.

In order to evaluate the cytotoxic potential of the treatments upon HT – 29 colon cancer cells, we investigated LDH activity in the culture media after 2, 6, and 24 h of exposure to free 5-FU, unloaded PHBHV nanoparticles, and 5-FU loaded nanoparticles, using as reference an untreated control. The results obtained after the spectrophotometric measurements were statistically analyzed, graphically represented in Figure 6, and in full accordance with the MTT cell viability assay. After 2 h of treatment, no significant changes were observed between treated and untreated samples in any of the experimental conditions. Furthermore, the cytotoxic profile of the unloaded PHBHV particles revealed that the free micro-formulation did not exert any cytotoxic effect on HT – 29 colon cancer cells, while 5-FU loaded carriers significantly increased LDH activity in the culture media after 6 and 24 h (*p* < .0001) as compared with the untreated HT – 29 cells. Also, under treatment with 5-FU an increase in the LDH activity in the culture media was observed both after 6 h (*p* < .01) and after 24 h (*p* < .0001) of treatment.

## Discussion

Colorectal cancer represents a significant cause of mortality and morbidity worldwide, being the third most common cancer in men and the second in women (Siegel et al., 2016). It is defined as one of the most aggressive types of cancers, as 50–60% of the diagnosed patients develop metastases (Cutsem et al., 2006). Despite the substantial improvements in colorectal cancer therapy, the administration of fluoropyrimidine-based chemotherapy remains the standard procedure for colorectal cancer patient’s treatment. Among fluoropyrimidines, 5-fluorouracil has been the most commonly used agent employed either alone or in different combinations (Gramont et al., 2000; Cutsem et al., 2004). However, the 5-FU based treatment regimens present several disadvantages such as a short biological half-life (Diasio & Harris, 1989), unpredicted severe toxicity in case of DPD alterations (Harris et al., 1991) and strong side effects (Tournigand et al., 2006). To address all these limitations, the development of novel drug delivery systems that can support a slow and sustained release of 5-FU, prevent it's *in vivo* degradation and decrease its toxicity would significantly contribute in the improvement of the life expectancy and quality of life of colorectal cancer patients.

In this context, we focused on the preparation of new nano-scaled carriers based on the natural PHBHV polymer, emphasizing on the importance of rational nanocarrier design, i.e. of using an appropriate concentration of the organic phase and aqueous phase to affect the final size, morphology, and overall characteristics of the produced nanoparticles. The novel biodegradable and biocompatible nanocarriers were designed to efficiently encapsulate a widely used therapeutic agent in colorectal cancer treatment, namely 5-FU. The biological effects of the nanocarriers encapsulating this classical chemotherapeutic agent upon colorectal cancer were evaluated using the human colorectal cancer cell line HT – 29 as an *in vitro* biological model.

After a thoroughly reviewing literature regarding the synthesis of particles based on a binary mixture of solvents (Pich et al., 2006; Poletto et al., 2007; Weiss-Angeli et al., 2008; Kumari et al., 2010), we concluded that for our purposes the emulsion diffusion technique represented the most suitable protocol to evaluate in construct carriers able to carry and release 5-FU in the desired manner. The natural polymer PHBHV and the stabilizer agent PVA were chosen as main components for particle preparation. PHBHV is an attractive polymer in the biomedical field due to its biocompatibility and biodegradability (Vroman & Tighzert, 2009), and represents a smart choice for drug delivery systems construction as its degradation rate is slower compared to other biodegradable synthetic polymers such as polylactic acid (PLA) and poly(lactic-co-glycolic acid) (PLGA) (Amass et al., 1998). PVA is a surfactant commonly used as a stabilizing agent that possesses the capacity to reduce the interfacial tension between the two phases due to its amphiphilic character given by the hydroxyl groups. The stabilizer forms strong interactions with particle surfaces, which can lead to its irreversible adsorption resulting in film formation on droplets, which in turn are transformed into particles. This film introduces an increased mechanical stability in the formed particles (Galindo-Rodriguez et al., 2004).

By using different variations of the preparation protocol, it was clearly highlighted that both polymer concentration and stabilizer agent concentration could easily modify the size and overall morphology of the obtained particles. Moreover, the polymer concentration was found to have a significant impact on the final relative number of particles obtained. More specifically, high concentration of PHBHV led to the formation of a small relative number of microparticles, with large sizes between 1 and 7 microns. In contrast, decreasing the polymer concentration led to a significant increase in the final relative number of nanoparticles obtained displaying smaller size, with diameters between 100 nm and 1 micron. Interestingly, despite the fact that decreasing the organic phase concentration represented an experimental strategy to adjust particle size, the 2% PHBHV concentration was determined as an equilibrium concentration and lowering the PHBHV amount under this threshold no longer led to the formation of smaller particles. We concluded that the 2% w/v PHBHV concentration was definitively the most appropriate concentration and was therefore chosen for 5-FU encapsulation. Additionally, to determine the role of the stabilizer on the size and characteristics of the particles, a screening to determine the optimum PVA concentration was also performed. When particles were obtained starting from a 5% w/v PVA concentration, an irregular mass attributed to residual stabilizer was observed on the surface of the particles altering their spherical shape. Moreover, the excess of PVA gathered on the surface of the particles lead to a clear increase in carrier size. Generally, the use of high stabilizer concentration is mandatory to obtain small-sized particles since a sufficient number of stabilizer chains is required to form submicronic emulsion droplets. In this case, the PVA concentration screening revealed that the optimal stabilizer concentration was the lower concentration of 2% w/v PVA. This led to the preparation of small particles, with a spherical shape that was not entrapped within stabilizer residual mass when combined with the optimal concentration of PHBHV. The optimal results were achieved using a 2% w/v natural polymer PHBHV concentration with a 2% w/v stabilizer concentration (20 mg/ml). These parameters led to a reduction of interfacial tensions causing the expected mechanical and steric stabilization that dictates the synthesis of smaller particles.

The nanocarriers were constructed with a characteristic core shell-like structure, defined by the presence of a hydrophobic core formed by PHBHV and a hydrophilic shell formed by the PVA stabilizer macromolecules. Drug encapsulation was performed directly by 5-FU solubilization in the polyester solution, leading to an asymmetric distribution of the drug molecules within both the core and the shell of the carriers. The drug release profile together with the encapsulation study revealed that a large amount of 5-FU remained entrapped within the PHBHV carriers. More specifically, the drug release study revealed that 20.1% of the encapsulated drug was released in 42 h, with a fast release profile during the first 6 h. This asymmetric release profile may be attributed a fast release sustained predominantly by the drug molecules absorbed on the particle surface during the first 6 h, while the drug molecules physically entrapped within the stabilizer outer shell sustain a constant, slower release. The remaining drug most probably entrapped within the polyester core is expected to be released during biodegradation.

To evaluate the biological effects of the novel synthesized PHBHV nanocarriers, human adenocarcinoma HT – 29 cells were used as an *in vitro* model for colorectal cancer. The viability MTT assay revealed that the bare PHBHV particles slightly altered the HT – 29 cell viability, suggesting that the polymer itself exhibits a cytotoxic effect upon the cancer cells. However, this effect was only observed after 6 h of exposure, while long term exposure did not present any further significant changes on HT - 29 cell viability. Regarding the 5-FU loaded PHBHV nanocarriers treatment, it was clearly highlighted that this treatment regimen induces significant changes to HT – 29 cell viability in a time-dependent manner, the most dramatic decrease of the cellular viability being observed after the time exposure. At low time points, the cytotoxic effect of the 5-FU PHBHV nanoparticles is attributed to the drug molecules that are attached on the outer surface of the particles, as well as to the drug molecules entrapped in the outer shell of the capsules. The decreasing viability of the HT – 29 cells in a time-dependent manner can be attributed to steady inner core drug release upon nanoparticle uptake, stemming from the biodegradation of the natural polymer PHBHV. The cytotoxic profile of the PHBHV nanocapsules confirmed the observations from the MTT assay and offered a complete picture of the drug release process of 5 – FU from PHBHV nanocarriers.

In summary, the highly toxic therapeutic agent 5-FU was successfully incorporated within nanoparticles specifically constructed from the natural hydrophobic polymer PHBHV with the well-studied, commercially available stabilizer PVA in the desired drug carrying morphologies. We developed PHBHV nanoparticles by an emulsification-diffusion method, but we importantly also demonstrated the effectiveness of the PVA stabilizer and the influence of polyester concentration on particles morphology. These newly developed nanocarriers could act as an efficient drug against carcinoma cells providing a simple therapeutic approach that can be expected to overcome the present 5 – FU delivery strategies. The PVA-PHBHV core-shell particles were developed as suitable carriers to protect drug by degradation to increase the therapeutic required concentration. Thus, nanoparticles are expected to reach, accumulate into the tumor cells and release the entrapped 5-FU. Based on the cytotoxicity assays performed, we concluded that the carrier itself exerts insignificant toxic effects on cells, while the treatment with 5FU loaded PHBHV particles induced carcinoma cells death. Future studies will focus on enzymatic degradation mechanism followed by *in vivo* assays evaluating the nanoparticles efficiency against induced metastases in mice.

## References

[CIT0001] Amass W, Amass A, Tighe B (1998). A review of biodegradable polymers: uses, current developments in the synthesis and characterization of biodegradable polyesters, blends of biodegradable polymers and recent advances in biodegradation studies. *Pol Int* 47:89–144.

[CIT0002] André T, Boni C, Mounedji-Boudiaf L, et al. (2004). Oxaliplatin, fluorouracil, and leucovorin as adjuvant treatment for colon cancer. *N Engl J Med* 350:2343–51. 10.1056/NEJMoa032709 15175436

[CIT0003] Arkenau HT, Bermann A, Rettig K, et al. (2003). 5-Fluorouracil plus leucovorin is an effective adjuvant chemotherapy in curatively resected stage III colon cancer: long-term follow-up results of the adjCCA-01 trial. Ann Oncol 14:395–9. 12598344 10.1093/annonc/mdg100

[CIT0004] Bartoş A, Bartoş D, Szabo B, et al. (2016). Recent achievements in colorectal cancer diagnostic and therapy by the use of nanoparticles. Drug Metabol Rev 48:27–46. 10.3109/03602532.2015.1130052 26828283

[CIT0005] Casarin SA, Malmonge SM, Kobayashi M, Agnelli JAM (2011). Study on in-vitro degradation of bioabsorbable polymers poly (hydroxybutyrate-co-valerate) – (PHBV) and poly (caprolactone) – (PCL) %. *J Biomater Nanobiotechnol *2:207–215.

[CIT0006] Cheng J, Teply BA, Sherifi I, et al. (2007). Formulation of functionalized PLGA–PEG nanoparticles for in vivo targeted drug delivery. Biomaterials 28:869–876. 17055572 10.1016/j.biomaterials.2006.09.047 PMC2925222

[CIT0007] Cutsem EV, Hoff PM, Harper P, et al. (2004). Oral capecitabine vs intravenous 5-fluorouracil and leucovorin: integrated efficacy data and novel analyses from two large, randomised, phase III trials. Br J Cancer 90:1190. 15026800 10.1038/sj.bjc.6601676 PMC2409640

[CIT0008] Cutsem EV, Moiseyenko VM, Tjulandin S, et al. (2006). Phase III study of docetaxel and cisplatin plus fluorouracil compared with cisplatin and fluorouracil as first-line therapy for advanced gastric cancer: a report of the V325 study group. *J Clin Oncol* 24:4991–4997. 10.1200/JCO.2006.06.8429 17075117

[CIT0009] Diasio RB, Harris BEJCP (1989). Clinical pharmacology of 5-fluorouracil. Clin Pharmacokinet 16:215–237. 2656050 10.2165/00003088-198916040-00002

[CIT0010] Engin AB, Nikitovic D, Neagu M, et al. (2017). Mechanistic understanding of nanoparticles' interactions with extracellular matrix: the cell and immune system. Particle Fibre Toxicol 14:22. 10.1186/s12989-017-0199-z PMC5483305 28646905

[CIT0011] Ferreira LS, Trierweiler JO (2009). Modeling and simulation of nanoparticles formation process: a diffusive approach In: de Brito Alves R.M., do Nascimento C.A.O., Biscaia E.C., editors. *Computer aided chemical engineering*. UK: Elsevier, 999–1004.

[CIT0012] Galindo-Rodriguez S, Allémann E, Fessi H, Doelker EJPR (2004). Physicochemical parameters associated with nanoparticle formation in the salting-out, emulsification-diffusion, and nanoprecipitation methods. *Pharmaceutical Res* 21:1428–1439. 10.1023/b:pham.0000036917.75634.be 15359578

[CIT0013] Gombotz WR, Pettit DK (1995). Biodegradable polymers for protein and peptide drug delivery. Bioconjugate Chem 6:332–351. 10.1021/bc00034a002 7578352

[CIT0014] Gramont Ad, Figer A, Seymour M, et al. (2000). Leucovorin and fluorouracil with or without oxaliplatin as first-line treatment in advanced colorectal cancer. *J Clin Oncol* 18:2938–2947. 10944126 10.1200/JCO.2000.18.16.2938

[CIT0015] Gratton SEA, Ropp PA, Pohlhaus PD, et al. (2008). The effect of particle design on cellular internalization pathways. Proc Natl Acad Sci USA 105:11613–11618. 18697944 10.1073/pnas.0801763105 PMC2575324

[CIT0016] Harris BE, Carpenter JT, Diasio RB (1991). Severe 5-fluorouracil toxicity secondary to dihydropyrimidine dehydrogenase deficiency. A potentially more common pharmacogenetic syndrome. *Cancer* 68:499–501. 10.1002/1097-0142(19910801)68:3<499::aid-cncr2820680309>3.0.co;2-f 1648430

[CIT0017] He J, Pei L, Jiang H, et al. (2017). Chemoresistance of colorectal cancer to 5-fluorouracil is associated with silencing of the BNIP3 gene through aberrant methylation. J Cancer 8:1187–96. 28607593 10.7150/jca.18171 PMC5463433

[CIT0018] Hunter AC, Moghimi SM (2017). Smart polymers in drug delivery: a biological perspective. Polym Chem 8:41–51.

[CIT0019] Jain D, Bajaj A, Athawale R, et al. (2016). Surface-coated PLA nanoparticles loaded with temozolomide for improved brain deposition and potential treatment of gliomas: development, characterization and in vivo studies. Drug Delivery 23:989–1006. 10.3109/10717544.2014.926574 25026415

[CIT0020] Jemal A, Siegel R, Ward E, et al. (2008). Cancer statistics. CA Cancer J Clin 58:71–96. 10.3322/CA.2007.0010 18287387

[CIT0021] Katiyar SS, Muntimadugu TA, Rafeeqi AJ, Domb W, et al. (2016). Co-delivery of rapamycin- and piperine-loaded polymeric nanoparticles for breast cancer treatment. Drug Delivery 23:2608–16. 26036652 10.3109/10717544.2015.1039667

[CIT0022] Khalili H, Chan ATJDD (2012). Sciences, is diabetes a risk factor for colorectal cancer? Dig Dis Sci 57:1427–9. 22531890 10.1007/s10620-012-2175-7

[CIT0023] Khalili H, Gong J, Brenner H, et al. (2015). Identification of a common variant with potential pleiotropic effect on risk of inflammatory bowel disease and colorectal cancer. Carcinogenesis 36:999–1007. 26071399 10.1093/carcin/bgv086 PMC4573660

[CIT0024] Krook JE, Moertel CG, Gunderson LL, et al. (1991). Effective surgical adjuvant therapy for high-risk rectal carcinoma. *N Engl J Med* 324:709–15. 10.1056/NEJM199103143241101 1997835

[CIT0025] Kumari A, Yadav SK, Yadav SC (2010). Biodegradable polymeric nanoparticles based drug delivery systems. Colloids Surf B: Biointerfaces 75:1–18. 19782542 10.1016/j.colsurfb.2009.09.001

[CIT0026] Kuskov AN, Kulikov PP, Goryachaya AV, et al. (2017). Amphiphilic poly-N-vinylpyrrolidone nanoparticles as carriers for non-steroidal, anti-inflammatory drugs: in vitro cytotoxicity and in vivo acute toxicity study. Nanomedicine 13:1021–30, 27884639 10.1016/j.nano.2016.11.006

[CIT0027] Kwon GS, Furgeson DY (2007). 4 - Biodegradable polymers for drug delivery systems In: Jenkins M., ed. *Biomedical polymers*. UK: Woodhead Publishing, 83–110.

[CIT0028] Leong J, Chin W, Ke X, et al. (2018). Disease-directed design of biodegradable polymers: Reactive oxygen species and pH-responsive micellar nanoparticles for anticancer drug delivery. Nanomedicine 14:2666–77. 10.1016/j.nano.2018.06.015 30017961

[CIT0029] Lindsetmo R-O, Joh Y-G, Delaney CP (2008). Surgical treatment for rectal cancer: an international perspective on what the medical gastroenterologist needs to know. Wjg 14:3281–9. 18528924 10.3748/wjg.14.3281 PMC2716581

[CIT0030] Luss AL, Kulikov PP, Romme SB, et al. (2018). Nanosized carriers based on amphiphilic poly-N-vinyl-2-pyrrolidone for intranuclear drug delivery. *Nanomedicine (Lond)* 13:703–15. 10.2217/nnm-2017-0311 29629829

[CIT0031] Matsuda T, Yamashita K, Hasegawa H, et al. (2018). Recent updates in the surgical treatment of colorectal cancer. Ann Gastroenterol Surg 2:129–36. 29863145 10.1002/ags3.12061 PMC5881369

[CIT0032] Meyerhardt JA, Catalano PJ, Haller DG, et al. (2003). Impact of diabetes mellitus on outcomes in patients with colon cancer. *J Clin Oncol* 21:433–40. 10.1200/JCO.2003.07.125 12560431

[CIT0033] Mora-Huertas CE, Fessi H, Elaissari A (2010). Polymer-based nanocapsules for drug delivery. Int J Pharma 385:113–142. 10.1016/j.ijpharm.2009.10.018 19825408

[CIT0034] Neagu M, Constantin C, Tampa M, et al. (2016). Toxicological and efficacy assessment of post-transition metal (Indium) phthalocyanine for photodynamic therapy in neuroblastoma. Oncotarget 7:69718–32. 27626486 10.18632/oncotarget.11942 PMC5342510

[CIT0035] Neagu M, Piperigkou Z, Karamanou K, et al. (2017). Protein bio-corona: critical issue in immune nanotoxicology. Arch Toxicol 91:1031–48. 27438349 10.1007/s00204-016-1797-5 PMC5316397

[CIT0036] Pardini B, Kumar R, Naccarati A, et al. (2011). 5-Fluorouracil-based chemotherapy for colorectal cancer and MTHFR/MTRR genotypes. Brit J Clin Pharmacol 72:162–3. 21204909 10.1111/j.1365-2125.2010.03892.x PMC3141199

[CIT0037] Pich A, Schiemenz N, Corten C, Adler H-JP (2006). Preparation of poly(3-hydroxybutyrate-co-3-hydroxyvalerate) (PHBV) particles in O/W emulsion. Polymer 47:1912–1920.

[CIT0038] Pinto Reis C, Neufeld RJ, Ribeiro A, Veiga JF (2006). Nanoencapsulation I. Methods for preparation of drug-loaded polymeric nanoparticles. Nanomedicine 2:8–21. 17292111 10.1016/j.nano.2005.12.003

[CIT0039] Piperigkou Z, Karamanou K, Engin AB, et al. (2016). Emerging aspects of nanotoxicology in health and disease: from agriculture and food sector to cancer therapeutics. Food Chem Toxicol 91:42–57. 26969113 10.1016/j.fct.2016.03.003

[CIT0040] Poletto F, Jager E, Re M, et al. (2007). Rate-modulating PHBHV/PCL microparticles containing weak acid model drugs. Int J Pharma 345:70–80. 10.1016/j.ijpharm.2007.05.040 17604922

[CIT0041] Quinn JF, Whittaker MR, Davis TP (2017). Glutathione responsive polymers and their application in drug delivery systems. Polym Chem 8:97–126.

[CIT0042] Radu I-C, Hudita A, Zaharia C, et al. (2017). Poly(hydroxybutyrate-co-hydroxyvalerate) (PHBHV) nanocarriers for silymarin release as adjuvant therapy in colo-rectal cancer. *Frontiers in Pharmacology* 8:508. 10.3389/fphar.2017.00508 PMC5539237 28824432

[CIT0043] Saito N, Murakami N, Takahashi J, et al. (2005). Synthetic biodegradable polymers as drug delivery systems for bone morphogenetic proteins. Adv Drug Deliv Rev 57:1037–1048. 15876402 10.1016/j.addr.2004.12.016

[CIT0044] Siegel RL, Miller KD, Jemal A (2016). Cancer statistics, 2016. CA Cancer J Clin 66:7–30. 26742998 10.3322/caac.21332

[CIT0045] Taghizadehghalehjoughi A, Hacimuftuoglu A, Cetin M, et al. (2018). Effect of metformin/irinotecan-loaded poly-lactic-co-glycolic acid nanoparticles on glioblastoma: in vitro and in vivo studies. *Nanomedicine (Lond)* 13:1595–606. 10.2217/nnm-2017-0386 30028222

[CIT0046] Torchilin VP (2010). Passive and active drug targeting: drug delivery to tumors as an example In: Schäfer-Korting M., editor. Drug delivery. Berlin, Heidelberg: Springer Berlin Heidelberg, 3–53. 10.1007/978-3-642-00477-3_1 20217525

[CIT0047] Tournigand C, Cervantes A, Figer A, et al. (2006). OPTIMOX1: a randomized study of FOLFOX4 or FOLFOX7 with oxaliplatin in a stop-and-go fashion in advanced colorectal cancer-a GERCOR study. J Clin Oncol 24:394–400. 16421419 10.1200/JCO.2005.03.0106

[CIT0048] Viard M, Reichard H, Shapiro BA, et al. (2018). Design and biological activity of novel stealth polymeric lipid nanoparticles for enhanced delivery of hydrophobic photodynamic therapy drugs. Nanomedicine 14:2295–305. 30059754 10.1016/j.nano.2018.07.006 PMC8034484

[CIT0049] Vroman I, Tighzert L (2009). Biodegradable polymers. Materials 2:307–344.

[CIT0050] Wang S, Wang L, Zhou Z, et al. (2017). Leucovorin enhances the anti-cancer effect of bortezomib in colorectal cancer cells. Sci Rep 7:682. 28386133 10.1038/s41598-017-00839-9 PMC5429730

[CIT0051] Weiss-Angeli V, Poletto FS, Zancan LR, et al. (2008). Nanocapsules of octyl methoxycinnamate containing quercetin delayed the photodegradation of both components under ultraviolet a radiation. J Biomed Nanotechnol 4:80–89.

[CIT0052] Zaheer S, Pemberton JH, Farouk R, et al. (1998). Surgical treatment of adenocarcinoma of the rectum. Ann Surg 227:800–11. 9637543 10.1097/00000658-199806000-00003 PMC1191380

[CIT0053] Zhang L, Chen Z, Mao G, et al. (2016). Nanostructured lipid carriers, solid lipid nanoparticles, and polymeric nanoparticles: which kind of drug delivery system is better for glioblastoma chemotherapy? Drug Deliv 23:3408–3416. 27181462 10.1080/10717544.2016.1189465

[CIT0054] Zhao J, Stenzel MH (2018). Entry of nanoparticles into cells: the importance of nanoparticle properties. Polym Chem 9:259–72.

